# Expression of genes involved in lipid droplet formation (*BSCL2*, *SNAP23* and *COPA*) during porcine in vitro adipogenesis

**DOI:** 10.1007/s13353-016-0350-9

**Published:** 2016-04-23

**Authors:** Beata Kociucka, Tatiana Flisikowska, Dariusz Mróz, Izabela Szczerbal

**Affiliations:** 1 Department of Genetics and Animal Breeding, Poznan University of Life Sciences, Wolynska 33, 60-637 Poznan, Poland; 2 Chair of Livestock Biotechnology, Technische Universität München, München, Germany

**Keywords:** Adipocyte, Fatness, Mesenchymal stem cells, Obesity, Model organism, Pig

## Abstract

Adipogenesis is a complex process of fat cells development driven by the expression of numerous genes. Differentiation of progenitor cells into mature adipocytes is accompanied by changes in cell shape, as a result of lipid accumulation. In the present study, expression of three genes involved in lipid droplet formation (*SNAP23*, *BSCL2* and *COPA*) was evaluated during porcine adipogenesis. It was found that mRNA levels of *BSCL2* and *SNAP23*, but not *COPA*, increased during differentiation. Redistribution of SNAP23 protein to different cellular compartments was observed when comparing undifferentiated mesenchymal stem cells and differentiated adipocytes. The BSCL2 protein was found to be highly specific to cells with accumulated lipids, while COPA protein coated the lipid droplets. Obtained results indicated that the studied genes may be considered as candidates for fatness traits in pigs. Moreover, this study has shown that the porcine in vitro adipogenesis system provides a useful tool for the characterisation of novel genes involved in adipose tissue accumulation.

**Electronic supplementary material:**

The online version of this article (doi:10.1007/s13353-016-0350-9) contains supplementary material, which is available to authorized users.

## Introduction

Accumulation of adipose tissue is one of the most extensively investigated process in pigs due to its economic importance for meat production as well as from the biomedical point of view, since the pig is considered as an important animal model for human obesity (Switonski et al. 2010). Adipose tissue expansion is a result of two processes — generation of new adipocytes (hyperplasia) and increasing the volume of existing adipocytes (hypertrophy) (Jo et al. 2009). In adipocytes, excess of circulating fatty acids, converted to triglycerides is stored within lipid droplets (LDs) (Rutkowski et al. 2015). The importance of insight into mechanisms regulating LD formation and expansion has been particularly stressed in recent years in the context of development of metabolic diseases, including obesity (Konige et al. 2014). Studies on the genetic background of fatness have revealed a number of genes involved in different cellular processes and pathways, among which genes controlling adipogenesis and lipid metabolism have been most extensively studied (Szczerbal and Chmurzynska 2008; Szczerbal et al. 2007).

Many experiments concerning adipocyte differentiation were performed on murine cell culture models (Rosen and MacDougald 2006) but species-specific regulation of adipogenesis justify such studies in the pig (McNeel et al. 2000). Porcine mesenchymal stem cells (pMSC) have been used for establishing in vitro systems of differentiation into adipocytes (Szczerbal et al. 2009; Casado et al. 2012; Lee et al. 2015; Bionaz et al. 2015). Adipogenesis in pigs, similarly to other organisms, is governed by a complex network of transcriptional factors, including the master regulator the peroxysome proliferator-activated receptor γ (PPARγ) and members of the CCAAT/enhancer binding protein (C/EBP) family (Boone et al. 1999). In addition, many other porcine pro- and anti-adipogenenic regulators, including miRNAs and lncRNAs have been characterised (Li et al. 2013; Pang et al. 2014; Jiang et al. 2015; Wei et al. 2015). System of in vitro differentiation into adipocytes is also a useful tool in studies on genetic regulation of lipid metabolism. Expression profile of genes regulating fatty acid synthesis, transport, storage and modifications (e.g. *FABPs*, *ACACA*, *LPL*, *SCD*, *FASN*) were analysed during porcine adipogenesis (Samulin et al. 2008, 2009). Some of these genes have been recognised as candidates for the accumulation of adipose tissue in pigs (Stachowiak et al. 2013; Bartz et al. 2013). Genes involved in lipid droplets formation are other candidate genes for porcine adiposity.

In the present study we analysed gene expression and protein cellular distribution for *SNAP23* (synaptosomal-associated protein 23), *BSCL2* (Berardinelli-Seip congenital lipodystrophy 2 - seipin) and *COPA* (coatomer protein complex α) during porcine in vitro adipogenesis. These genes were selected based on their known function in lipid droplet formation — the *SNAP23* is involved in increasing the size of lipid droplets (Boström et al. 2010), the *COPA* is a subunit of COPI complex involved in the growth of lipid droplets (Wilfling et al. 2014) and the *BSCL2* encodes seipin, which is important for lipid droplet biogenesis and morphology (Cartwright et al. 2015). We hypothesised that an increased accumulation of lipid droplets during adipogenesis may correlate with increased expression of these genes. Moreover, changes of cellular morphology observed during differentiation of mesenchymal stem cells into adipocytes may result in relocalisation of the selected proteins to different cellular compartments.

## Materials and methods

### pMSC culture and induction of adipogenesis

Porcine mesenchymal stem cells isolated from bone marrow (BM) of Polish Large White pig were cultured in DMEM medium (Gibco) supplemented with 10 % FCS (Sigma), 5 ng/ml FGF-2 (PromoKine), 1 × NonEssential amino acids (Gibco), 2 mM L-Glutamine (PAA), 1 mM 2-Mercaptoethanol (Sigma) and a mixture of antibiotics (100 U/ml of Penicillin, 100 μg/ml of Streptomycin, Sigma) at 37 °C in 5 % CO2. In all experiments pMSC were used at early passages (P4-P8). To induce adipogenic differentiation, pMSCs were grown to confluency and were cultured with adipogenic differentiation medium composed of the basal medium supplemented with 50 μM IBMX (Sigma), 1 μM Dexamethason (Sigma-Aldrich), 100 μM Indomethacin (Sigma-Aldrich), 1 × ITS and 1 × Linoleic Acid (Sigma -Aldrich) and FGF-2 (5 μg/ml)). The cells were cultured for 7 days and adipocyte differentiation was monitored by phase contrast microscopy.

### Indirect immunofluorescence

For immunofluorescence staining, cells cultured on glass cover slips were fixed in 4 % paraformaldehyde in PBS (w/v) for 10 min at room temperature, followed by washing in PBS. The cells were then treated with 0.1 % Triton X-100 in PBS (v/v) for 5 min, washed three times in PBS and blocked for 30 min in 3 % bovine serum albumin (w/v). Primary rabbit polyclonal antibodies (Abcam) against the SNAP23, BSCL2 or COPA proteins were used. Specificity of antibodies was verified by western blot analysis. The cells were incubated overnight with primary antibodies in 1:100 dilution at 4 °C. After three washes in PBS cells were incubated for 1 h with a secondary antibody — goat anti-rabbit Alexa Fluor 594 (Invitrogen), diluted 1:200. After further washing in PBS cells were stained with BODIPY dye (Invitrogen) to visualise lipid droplets. Finally, nuclei were counterstained with DAPI in the Vectashield medium (Vector Laboratories).

### Microscopy and image analysis

Cells were examined under a fluorescence microscope (E600 Eclipse, Nikon) and a confocal laser scanning microscope (LSM 510Meta, Zeiss) equipped with three lasers: HeNe 543 nm, Argon 488 nm and Diode 405 nm. The filters were 560 nm for Alexa Fluor 594, 505 nm for BODIPY dye and 420 nm for DAPI. Images from a confocal microscope were taken through a Plan Apo oil immersion objective 100×/1.4NA or Plan Neofluar oil immersion objective 40×/1.3NA using Zeiss LSM 510 vs 3.2 SP1 software. Stack of optical sections with an axial distance of 0.4 μm were collected. Objectives, the pinhole and filters were kept constant throughout the experiment. The Zeiss LSM Image Browser software was used for image analysis. Amounts of lipids accumulated in pMSCs (day 0) and during 3, 5 and 7 days of adipogenic differentiation were measured using the fluorescence intensity parameter derived from the BODIPY dye (green fluorescence) using the ImageJ software.

### Quantitative real-time PCR

Total RNA extraction from pMSC (day 0 of adipogenesis differentiation) and from differentiated cells (1–7 days) was performed in triplicate using the TriPure Isolation Reagent RNA (Roche) according to the standard protocol. Concentration and purity of RNA was determined using a Nanodrop Spectrophotometer. For cDNA synthesis, an aliquot of 2 μg RNA was reversely transcribed using the Transcriptor High Fidelity cDNA Synthesis kit (Roche). Primer sets for quantitative real-time PCR of the studied and reference genes (Table S1) were designed using PRIMER 3 software (http://simgene.com/Primer3). The relative quantification of the mRNA level was performed in duplicates based on the Second Derivative Maximum Method on a capillary real-time PCR LightCycler 2.0 (Roche) using the Fast Start DNA Master Plus SYBR Green I kit (Roche). Standard curves were designed as 10-fold dilutions of the PCR products. Relative transcript levels of the studied genes were calculated after correction via the transcript level of a reference gene — cyclophilin A (*PPIA*), which has shown stability during adipogenic differentiation.

### Statistical analysis

Relative transcript levels of the *BSCL2*, *SNAP23* and *COPA* genes presented as mean ± SEM were analysed using SigmaPlot (Version 11.0.1, Systat Software). Statistical analyses for differences during adipogenesis were performed using one-way ANOVA followed by the Holm-Sidak post hoc test. Statistical significance was set at *P* ≤ 0.05.

## Results and discussion

**Fig. 1:**
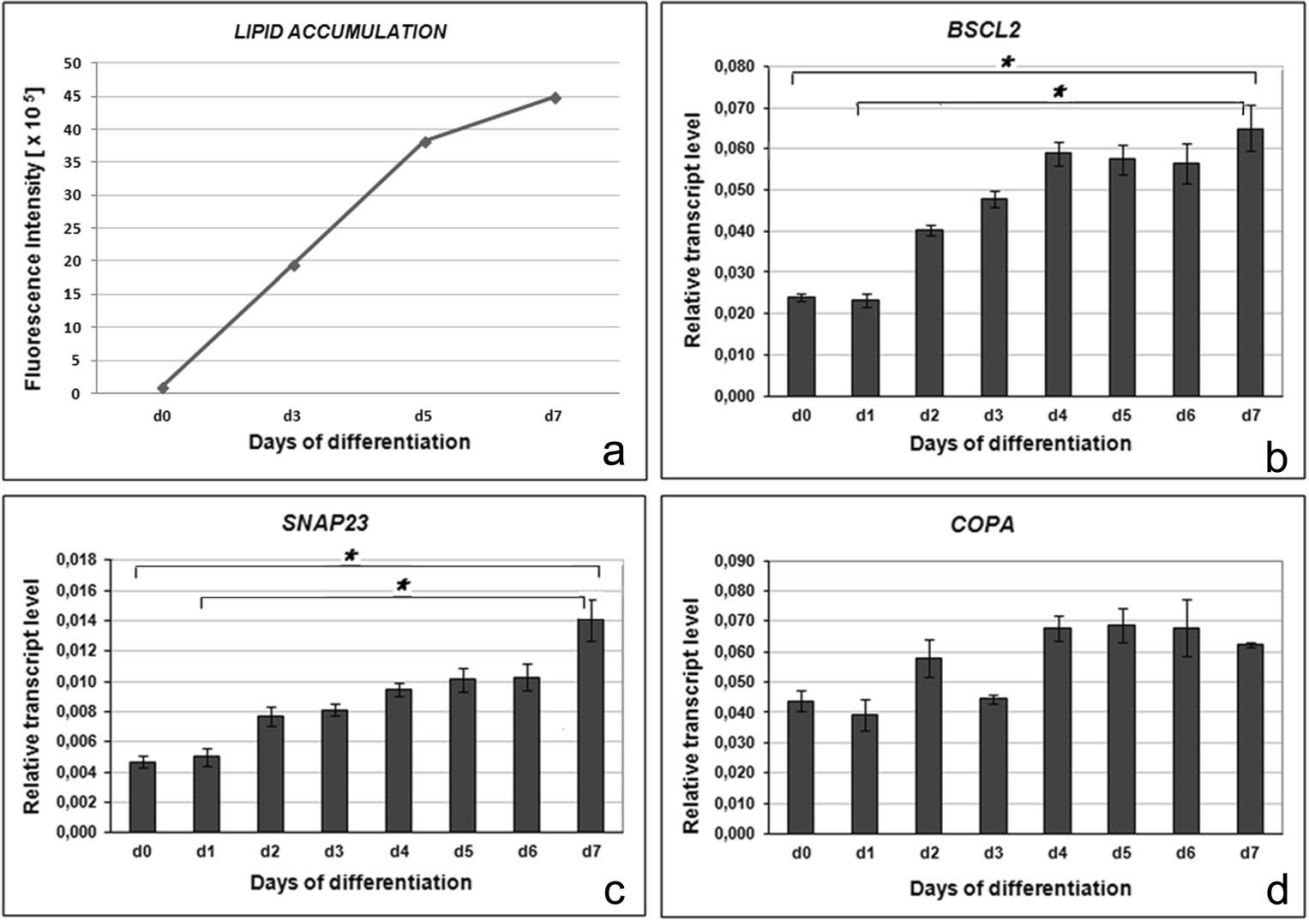
Monitoring of lipid accumulation and transcript levels of studied genes during 7-day adipogenesis. (**a**) Accumulation of lipids is presented as an increase of green fluorescence intensity after BODIPY staining. Relative mRNA levels (mean ± SEM) of *BSCL2* (**b**), *SNAP23* (**c**) and *COPA* (**d**) genes. Statistically significant differences are indicated by asterisks (*P* < 0.05)

Transcript levels of the *BSCL2*, *SNAP23* and *COPA* genes were analysed for 7 days of differentiation of porcine mesenchymal stem cells into adipocytes. Lipid accumulation, monitored with the use of the BODIPY staining, showed an increasing amount of lipid droplets during successive days of adipogenesis (Fig. 1a). Transcripts of all studied genes were detected in undifferentiated cells (day 0). The relative transcript level of *SNAP23* was lower than those of the *BSCL2* and *COPA* genes. Interestingly, mRNA levels of the *BSCL2* and *SNAP23* genes showed an upward trend from day 2 of adipogenesis and reached the highest level on day 7 (Fig. 1b, c). The significant differences (*P* < 0.05) have been observed between day 0 and day 7, as well as between day 1 and day 7. In contrast, expression of the *COPA* gene was uniform and no association between lipid droplet accumulation and mRNA abundance was found (Fig. 1d).

**Fig. 2:**
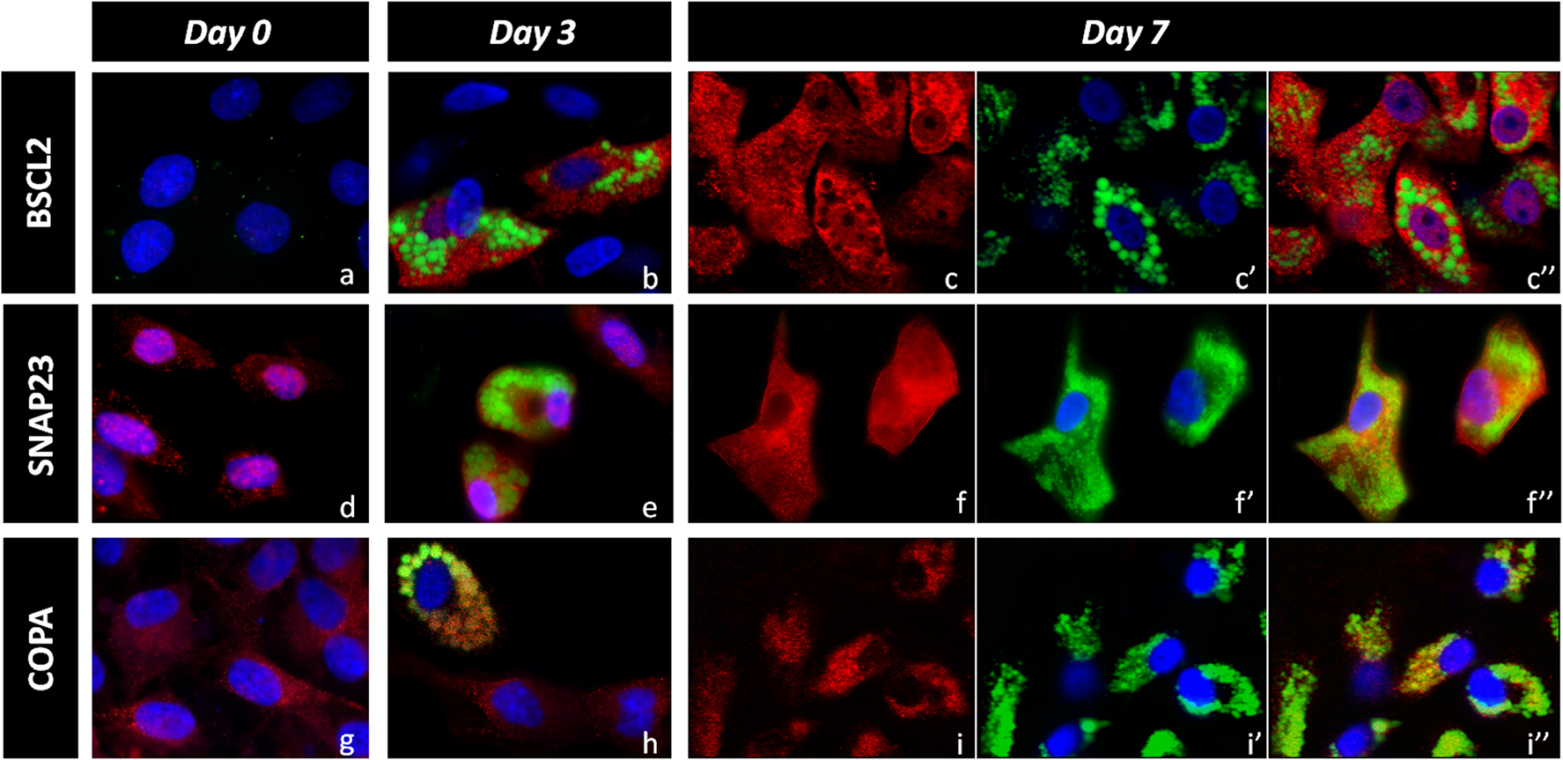
Representative images of immunolocalisation of BSCL2 (**a**, **b**, **c**), SNAP23 (**d**, **e**, **f**) and COPA (**g**, **h**, **i**) proteins at day 0, 3 and 7 of adipocyte differentiation. Proteins were detected with a specific antibody (*red*), lipids were stained with BODIPY dye (*green*) and nuclei were counterstained with DAPI (*blue*). Merged images are shown for day 0 and 3, while at day 7 protein (**c**, **f**, **i**) and lipids/nuclei (c’, f’, I’) are shown separately and as merged images (c”, f”, I”)

To examine the cellular location of the protein encoded by the analysed genes we performed immunofluorescent staining on fixed cells from days 0, 3 and 7 of porcine adipogenesis. While, SNAP23 and COPA proteins were detected from day 0 of the differentiation, the BSCL2 protein was not found in undifferentiated mesenchymal stem cells. The BSCL2 protein was preferentially located in cytoplasm and was highly specific to cells with accumulated lipid droplets (Fig. 2a–c), but no differences were observed in terms of its distribution to specific cellular compartments. The SNAP23 has been found in the cytosol and was preferentially located around the cell nucleus in undifferentiated mesenchymal stem cells. At day 3 and 7 of adipogenesis the protein was more evenly distributed in the cytosol, but its elevated accumulation was detected in the plasma membrane (Fig. 2d–f). The COPA protein was distributed in the cytoplasm of both undifferentiated and differentiated cells but when lipid droplets started to appear, more intense signals were observed on their LD surface (Fig. 2g–h).

The *BSCL2* gene encodes the seipin protein, involved in the regulation of adipocyte differentiation and lipid droplets formation. *BSCL2* mutations are responsible for congenital lipodystrophy type 2 in humans (Chen et al. 2012). Studies on mice and humans have shown that the *BSCL2* expression was increased significantly during adipocyte differentiation (Payne et al. 2008). Moreover, it has been found that expression of this gene is not crucial for lineage commitment in murine C3H10T1/2 mesenchymal stem cells but is up-regulated during late stages of differentiation (Chen et al. 2009). These findings are in agreement with the results of the present study showing an increase of the transcript level during adipogenesis in pigs. Since seipin has been recognised as a endoplasmic reticulum-resident protein, we found an intense immunostaining in the cytoplasm, which was highly specific to cells accumulating lipids. The protein was not detected in mesenchymal stem cells, although the transcript of this gene was present. Most likely the BSCL2 protein expression was too low for immunodetection. Interestingly, it was revealed that the BSCL2 has a different function depending on cell type — it promotes lipid droplets formation in adipocytes and prevents it in other cells (Yang et al. 2014).

Lipid droplet size is increased through fusion of primordial droplets and SNARE proteins, including the SNAP23, are involved in this process (Boström et al. 2007). Information concerning transcript levels of the *SNAP23* gene during adipogenesis is limited. In our study we found an increase of *SNAP23* transcript level in late stages of adipocyte differentiation. On the other hand, the level of SNAP23 protein remained constant during differentiation of the 3T3-L1 cell line (Torrejón-Escribano et al. 2002). It has been reported that the SNPA23 function can be regulated through its redistribution to different cell compartments. Torrejón-Escribano et al. (2002) observed relocation of this protein from the perinuclear region towards the plasma membrane during adipocyte differentiation. In contrast, in cardiomyocytes treated with fatty acids, as well as in skeletal muscle of patients with type 2 diabetes, the protein moved from the plasma membrane to the cell interior (Boström et al. 2007, 2010). During porcine adipogenesis, similar to what was reported in humans by Torrejón-Escribano et al. (2002), we observed an accumulation of the protein near the nucleus at day 0 of adipogenesis, while at day 3 and 7 a more diffused location in the cytosol and an elevated level in the plasma membrane were found. This finding indicates relocation of this protein during porcine adipogenesis.

The COPA protein is a subunit of the coatomer protein I (COPI) complex, which is involved in the regulation of lipid droplet formation by establishing connections between LDs and the endoplasmic reticulum, by allowing relocalisation of enzymes involved in triacylglycerol metabolism (Wilfling et al. 2014). A study on genes encoding COPI subunits, including COPA, showed that targeting these genes by siRNA led to increased lipid accumulation (Beller et al. 2008). Thus, we assumed that these genes could have a crucial role in lipid droplet formation. However, we did not observe any association between the transcription profile of *COPA* and the amount of accumulated lipids during differentiation. We also found that COPA protein is located on the surface of LDs, as was previously shown for other proteins from the COPI pathway (Wilfling et al. 2014).

In vitro differentiation of mesenchymal stem cells into mature adipocytes is a useful model for functional studies on candidate genes for fatness traits in pigs. The presented results, obtained in the in vitro system, should be treated as an introduction to further studies on fat tissue samples collected from growing pigs. We assumed that morphological changes that occur during adipogenesis, may be associated with alterations in gene expression and cellular location of proteins involved in LDs formation. Relocation of key enzymes for lipid metabolism and proteins associated with LDs to different cellular compartments is a well known mechanism having functional significance in many processes, including lipid droplets growth (Jacquier et al. 2011; Wilfling et al. 2013). Indeed, we observed differences in the transcript level of *BCLS2* and *SNAP23* genes as well as a specific pattern of distribution of the analysed protein during porcine adipogenesis. Since these genes are important for adipocyte differentiation it can be anticipated that they may also may play a role in pig adiposity. Identification of novel candidate genes involved in lipid droplet formation will provide a better understanding of the molecular mechanism responsible for adipose tissue accumulation in pigs.

## Electronic supplementary material

Below is the link to the electronic supplementary material.

Table S1 (DOCX 14 kb)
