# Supplementary material for: Expression of genes involved in lipid droplet formation (BSCL2, SNAP23 and COPA) during porcine in vitro adipogenesis
Source: J Appl Genet. 2016 Apr 23;57(4):505–10. doi: 10.1007/s13353-016-0350-9 (PMC5061828; doi:10.1007/s13353-016-0350-9)
Supplement: Supplementary file 1 — (DOCX 14 kb) [file 13353_2016_350_MOESM1_ESM.docx]

**Supplementary table**

**Table S1.** Real-time PCR primer sequences for studied and reference genes.

| **Gene** | **Primer sequence ^1^** | **Product length (bp)** | **GenBank acc. no./ reference** |
| --- | --- | --- | --- |
| ***SNAP23*** | F: 5’AAGTGCTGTGGCCTTTGTGT3’  R: 5’CATTTCGTCTTCTCTGGCATC3’ | 225 | NM_001244719 |
| ***BSCL2*** | F: 5’GCTCTGGGTGTCTGTCTTCC3’  R: 5’AGCTCTAAGGTGACGCGGTA3’ | 213 | NM_001128459 |
| ***COPA*** | F: 5’GGAGTCGGATGTGAGAGGAA3’  R: 5’CGAGGGTGGAAGACAGCA3’ | 249 | XM_001928697 |
| ***PPIA*** | F:5’CACAAACGGTTCCCAGTTTT3’  R: 5’TGTCCACAGTCAGCAATGGT3’ | 171 | Stachowiak et al., 2013 |

^1^ F = forward; R = reverse.
